# Gait Stability and Its Influencing Factors in Older Adults

**DOI:** 10.3389/fphys.2018.01955

**Published:** 2019-01-24

**Authors:** Daniel Hamacher, Dominik Liebl, Claudia Hödl, Veronika Heßler, Christoph K. Kniewasser, Thomas Thönnessen, Astrid Zech

**Affiliations:** ^1^Institute of Sport Science, Friedrich Schiller University of Jena, Jena, Germany; ^2^Department of Statistics, University of Bonn, Bonn, Germany

**Keywords:** balance, muscular fitness, physical activity, pain, peripheral sensation, gender, proprioception, gait variability

## Abstract

A stable gait pattern is a prerequisite to successfully master various activities of daily living. Furthermore, reduced gait stability is associated with a higher risk of falling. To provide specific intervention strategies to improve gait stability, gaining detailed knowledge of the underlying mechanism and influencing factors is of utmost importance. The effects of relevant influencing factors on gait stability are poorly examined, yet. Therefore, the aim of the current study was to quantify the effects of various influencing factors on gait stability. In a cross-sectional study, we assessed dynamic gait stability and relevant influencing factors in 102 older adults (age >65 years). In addition to dynamic gait stability (largest Lyapunov exponent [LLE] and gait variability measures) during normal over-ground (single-task: ST) and dual-task (DT) walking, we registered the following influencing factors: health status (SF12), pain status (painDETECT, SES), fear of falling (falls efficacy scale), depression (CES-D), cognition performance (Stroop test), physical activity (Freiburger Fragebogen zur körperlichen Aktivität), proprioception (joint position sense), peripheral sensation (mechanical and vibration detection threshold), balance performance (static balance on force plate) and muscular fitness (instrumented sit-to-stand test). We used a principal components regression to link the identified principal components with the gait stability and gait variability responses. The four principal components “strength and gender” (e.g., *p* = 0.001 for LLE during ST), “physical activity” (e.g., *p* = 0.006 for LLE during ST), “pain” (e.g., *p* = 0.030 for LLE during DT) and “peripheral sensation” (e.g., *p* = 0.002 for LLE during ST) were each significantly associated with at least two of the analyzed gait stability/variability measures. The dimension “balance” was a significant predictor in only one gait measure. While “proprioception” tends to correlate with a gait variability measure, we did not find a dependency of mental health on any gait measure. In conclusion, the participants' ability to recover from small perturbations (as measured with the largest Lyapunov exponent) seems to be related to gender and strength, the amount of physical activity the participants spent every week, peripheral sensation and pain status. Since the explained variance is still rather low, there could be more relevant factors that were not addressed, yet.

## Introduction

Falls in the elderly occur quite frequently. 30–60% of older adults fall at least once a year (Rubenstein, 2006; Inouye et al., 2009) and up to 20% of falls result in injuries (Rubenstein, 2006), hospitalization or death in older adults (Rubenstein, 2006; Pfortmueller et al., 2014). Western health care systems spend 0.85 to 1.5% of their total health care expenditures (Heinrich et al., 2010) on the consequences of falls, rendering this issue socio-economically relevant. Considering the effect on the individual's quality of life, the prevention of falls is of utmost importance.

Risk factors for falls and fall-related injuries can be categorized into (I) environmental risk factors, (II) behavioral risk factors, (III) biological risk factors, and (IV) socioeconomic risk factors. However, besides standing quietly and sitting down, walking is one of the most common daily activities leading to falls (Robinovitch et al., 2013). Thus, it is not surprising that an unstable gait pattern is associated with a higher risk of falling in older adults (Hamacher et al., 2011; Bruijn et al., 2013). In addition, walking is a prerequisite to handle a wide range of daily activities (Bramble and Lieberman, 2004). Since gait measures are predictors of mobility (Brach et al., 2007), a stable gait also ensures social participation not only in the elderly. This highlights the importance of a stable gait in various contexts.

According to the literature, age is a strong predictor of falls (Lord et al., 2003; Ambrose et al., 2013; Pfortmueller et al., 2014) as well as of an unstable gait (Terrier and Reynard, 2015). Other (predominantly age-related) intrinsic determinants are frequently discussed as risk factors for falls. These intrinsic determinants include gender, muscle strength or muscle power, balance, peripheral sensation (proprioception, vibration sense, tactile sensitivity), cognition, and diseases, such as diabetes mellitus (Lord et al., 2003; Ambrose et al., 2013; Pfortmueller et al., 2014). Even depression seems to be a factor related to an altered gait pattern and the probability of falling (Paleacu et al., 2007; Ambrose et al., 2013). Furthermore, gait characteristics (Ambrose et al., 2013) and in particular gait variability and gait stability measures (Hamacher et al., 2011; Bruijn et al., 2013) or the ability to dual-task during gait (Ambrose et al., 2013) are also highly associated with the risk of falling. Although this may indicate interactions between the above-mentioned person-related determinants and walking abilities in older adults, the effects of intrinsic factors on gait variability or gait stability itself were barely reported in the literature. Since knowledge of relevant factors affecting a stable gait is a prerequisite to (1) develop and to evaluate specific intervention strategies, (2) improve gait stability, (3) reduce the number of falls, or (4) ensure save social participation in older adults, we aimed to assess the effects of intrinsic factors associated with falling on gait stability and gait variability as well as on dual-task walking in older adults.

In general, exercise interventions are capable to improve gait variability (Wollesen et al., 2015) and, furthermore, an improved gait pattern enhances physical functioning, physical activity and social participation (VanSwearingen et al., 2011). However, to be able to deduce individually adjusted fall prevention programs (Pfortmueller et al., 2014) that are more efficient and to better understand the underlying mechanisms of gait stability, influencing factors must be identified.

In a broader sense, a stable gait is a gait pattern that does not lead to falls. There are different types of gait stability (Bruijn et al., 2013): for example, dealing with small internal (e.g., neuromuscular noise) and external perturbations (e.g., surface friction) during normal overground walking or recovering from larger perturbations (e.g., a trip or a slip). This study will focus on the former type. Here, regarding fall risk, variability measures and the largest Lyapunov exponent depict the best construct, predictive, and convergent validity (Bruijn et al., 2013) and are, thus, chosen for this study.

Therefore, the aim of the current study was to explore the effect of all above-mentioned intrinsic risk factors on gait stability and gait variability during normal and dual-task walking in older adults. Additionally, factors that interfere with a stable gait, e.g., pain (Hamacher et al., 2016), osteoarthritis and having a joint replacement (Yakhdani et al., 2010; Hamacher, 2014), were also considered.

## Materials and Methods

### Study Design and Participants

In a cross-sectional study, 102 (52 female and 50 male) healthy older adults with a mean age of 72 years (SD = 4.5 years) and a mean body mass index of 27 (SD = 3.6) were recruited using a newspaper announcement. Inclusion criteria were an age of at least 65 years and the ability to walk for 10 min without any aids. Acute neurological, orthopedic or cardiovascular diseases lead to exclusion. Participants with common age-related diseases, such as diabetes mellitus, osteoarthritis, high blood pressure or an implanted prosthesis, were included in the study. This study was carried out in accordance with the recommendations of the Declaration of Helsinki with written informed consent from all subjects. The protocol was approved by the Ethical Commission of the Faculty of Social and Behavioral Sciences, Friedrich Schiller University of Jena (no. FSV 16/05).

### Testing Procedure

Each participant came at 2 different days within 2 weeks to complete the tests. At the first test day, a standardized gait analysis was conducted. At the second test day, influencing factors were registered. In total, all tests lasted 3.5 h. A summary of all outcomes is given in Tables 1, 2.

**Table 1:** Overview of the dependent variables.

**Dependent variables**			**Abbreviation**
Primary analysis	Gait stability	Local dynamic stability (LDS) of the foot during single-task (ST) walking	LDS_foot, ST_
		Local dynamic stability (LDS) of the foot during dual-task (DT) walking	LDS_foot, DT_
		Local dynamic stability (LDS) of the trunk during single-task (ST) walking	LDS_trunk, ST_
		Local dynamic stability (LDS) of the trunk during dual-task (DT) walking	LDS_trunk, DT_
Secondary analysis	Gait variability	Stride-to-stride standard deviation (SD) of stride length during single-task walking	SD_StrideLength, ST_
		Stride-to-stride standard deviation (SD) of stride length during dual-task walking	SD_StrideLength, DT_
		Stride-to-stride standard deviation (SD) of stride time during single-task walking	SD_StrideTime, ST_
		Stride-to-stride standard deviation (SD) of stride time during dual-task walking	SD_StrideTime, DT_

**Table 2:** Overview of all independent variables.

**Variables**	**Abbreviation**	
Anamnesis	Age	
	Gender	
	Body mass index	BMI
	Osteoarthritis	
	Any kind of joint replacement	Prosthesis
Muscular fitness	Normalized Peak Power during the sit-to-stand test	S2S_PeakV_
	Normalized Mean Power during the sit-to-stand test	S2S_MeanV_
Balance	Sway during double-leg (dl) stance with eyes open (eo)	Sway_dl, eo_
	Sway during semi-tandem (st) stance with eyes open (eo)	Sway_st,eo_
	Sway during double-leg stance (dl) with eyes closed (ec)	Sway_dl,ec_
	Sway during semi-tandem stance (st) with eyes closed (ec)	Sway_st,ec_
Pain	Neuropathic component (painDETECT)	Pain_neuro_
	Affective component (SES)	Pain_affect_
	Sensory component (SES)	Pain_sens_
	sub-scales rhythmicity of the sensory component (SES)	Pain_sens, rhythm._
	sub-scales local depth of the sensory component (SES)	Pain_sens, depth_
	sub-scales temperature of the sensory component (SES)	Pain_sens, temp._
Cognition	Time needed for incongruent stimulus condition (Stoop test)	Cog_ink_
	Time costs for the incongruent stimulus (compared to the ink-naming condition, Stroop test)	Cog_relink_
Fear of Falling	FES-I score	FES-I
Depression	CES-D score	CES-D
Health status	Physical component summary score of the SF-12	SF12p_hysical_
	Mental component summary score of the SF-12	SF12m_ental_
Peripheral sensation	Vibration detection threshold of the “quantitative sensory testing” battery	Sens_VibDT_
	Mechanical detection threshold (MDT) of the “quantitative sensory testing” battery	Sens_MechDT_
Proprioception	Mean of the absolute error of an active/active angle reproduction test	Prop_MeanErr_
	Standard deviation of the error of an active/active angle reproduction test	Prop_SDErr_
Physical activity	Total sum of physical activity (FFkA questionnaire)	FFkA_total_
	Basic (common daily activities) physical activity (FFkA questionnaire)	FFkA_basic_
	Extracurricular physical activity (FFkA questionnaire)	FFkA_Extracurr_
	Sports activity (FFkA questionnaire)	FFkA_Sports_

#### Anamnesis

To check the inclusion and exclusion criteria, diseases, motor-functional complaints, and medication were registered. The participants were explicitly asked if they have diabetes mellitus, osteoarthritis in any joint of the lower extremities or any kind of prosthesis at the lower extremities. To be able to better describe our cohort, we also asked the participants how frequently they have fallen (while walking or standing) within the last 12 months.

#### Gait Analysis

To assess gait parameters, a standardized gait analysis was conducted in an empty sports hall. Thereto, inertial sensors (MTw2, Xsens Technologies B.V., Enschede, The Netherlands, range of measurement of angular velocity: ±1,200 deg/s, sampling rate: 100 Hz) were attached to the dominant forefoot with tape and to the thorax (strap underneath the arms, thus sensor at upper thoracic spine) with an elastic strap. The dominant foot was identified using the German version of the Lateral Preference Inventory (Ehrenstein and Arnold-Schulz-Gahmen, 1997). To improve the reliability of gait measures, the participants walked on a 25 m track up and down once with their comfortable walking pace to familiarize to the test setup (Hamacher et al., 2017). Thereafter, the participants completed the following conditions in randomized and balanced order: (a) Motor single-task condition: walking up and down the 25 m track with their comfortable walking pace for 4 min; (b) Dual-task condition: Walking with comfortable walking pace while reciting serial three subtractions (starting from a random three-digit number) for 4 min.

From the kinematic walking time series, the following gait parameters were calculated: stride length and stride time as well as the intra-individual stride-to-stride variability (standard deviations) of stride length and stride time as measures of gait variability. The reliability of the measurement system is verified (Hamacher et al., 2014).

As a measure of local dynamic gait stability (LDS), the short-time largest Lyapunov exponent was determined for foot and trunk kinematics separately using an evaluated algorithm (Hamacher et al., 2015). Since for normal overground walking, the highest effects comparing young vs. older adults were observed when analyzing time series derived from three-dimensional angular velocity data of the foot (Hamacher et al., 2015), we used those time series, too. For each participant, the three-dimensional angular velocity data of 100 strides were time-normalized to 10,000 samples, resulting in an average of 100 samples per stride. Thereafter, a state space was built upon on the time-normalized data using the method of time-delayed embedding. The time delay and embedded dimension were chosen based on the first minimal mutual information (Fraser and Swinney, 1986) and the false nearest neighbors analysis (Kennel et al., 1992), respectively. A fixed time-delay τ (mean across all participants: τ_foot_ = 9, τ_trunk_ = 11) and embedded dimension dE (maximum across all participants: dE_foot_ = dE_trunk_ = 12) was used for all participants. The largest Lyapunov exponent was calculated using Rosenstein and coworkers' algorithm (Rosenstein et al., 1993). Thereto, the Euclidean distances of each point in state space of initially nearest neighbors were tracked in time and the mean of the logarithm of this divergence curves was calculated. The largest Lyapunov exponent was defined as the slope of the linear fit through approximately 0–0.5 strides. Larger values indicate lower local dynamic gait stability. The largest Lyapunov exponent quantifies the ability of a dynamic system (human gait) to recover from small perturbations (Bruijn et al., 2013).

Compared to the gait variability measures, the largest Lyapunov exponent depict a slightly better construct validity (Bruijn et al., 2013). Therefore, the outcomes local dynamic stability (LDS) of trunk and foot during single-task and dual-task walking were considered primary criterions to be predicted by the assumed influencing outcomes. In a secondary analysis, the gait variability parameters SD of stride length and SD of stride time during single-task and dual-task walking were analyzed.

#### Muscular Fitness

To measure muscular power of the lower extremities, the sit-to-stand transfer has already been successfully conducted in older adults (Lindemann et al., 2003; Zech et al., 2011; Zijlstra et al., 2012). We used an instrumented version to assess muscular power (Zijlstra et al., 2010). Thereto, an inertial sensor (MTw2, Xsens Technologies B.V., Enschede, The Netherlands, sampling rate: 100 Hz) was fixed to the back of the pelvis. Compared to a force plate based approach, power calculated from inertial sensor data fixed to the pelvis depict high correlations (*r* = 0.95 for fast movements, Zijlstra et al., 2010). The participants were placed on the front part of a chair (height: 0.47 m, no armrests, arms crossed over the chest). They were asked to stand up as fast as possible. The participants were asked to sit/stand motionless immediately before and after the sit-to-stand transition. As described below, this was used as a boundary condition for parameter calculation.

Using the sensors orientation (quaternions) and the three-dimensional accelerometer data, the vertical component of the acceleration data was extracted, and gravitational acceleration was removed by subtracting 9.81 m/s^2^. Vertical movement velocity was calculated by numerical integration (Heun's method). Prior to and after the sit-to-stand transition, the movement velocity is considered zero. Any deviations from zero (e.g., due to the numerical integration) were removed by subtracting a straight line which was fitted through two points: the pre-test and the post-test vertical velocity value. The Power was calculated as the arithmetic product of the vertical movement velocity, gravitational acceleration g, and the body weight. Power was then normalized to the subject's body weight. Based on the resulting power-time curve, peak power and mean power (mean power during the sit-to-stand transition where the vertical movement velocity was at least 0.1 m/s) were calculated as outcomes.

#### Balance

Balance performance was assessed during double-leg stance (feet together) and semi-tandem stance (toe of the dominant foot slightly touching the contralateral heel) on a force platform (type 9260AA6, Kistler Instrumente GmbH, Winterthur, Swiss). During the test, the participants did not wear shoes but socks. The two stance conditions were completed with eyes open and eyes closed for 30 s each stance condition. In all stance conditions, hands were held on hips. During the open eyes conditions, the participants were asked to look at a cross placed at eye level 1 m in front of the participant. With the aid of the MARS software (Kistler Instrumente GmbH, Sindelfingen, Germany) the mean velocity of the two-dimensional velocity of the center of pressure was calculated for each balance condition.

#### Pain

The pain status was assessed using the painDETECT questionnaire and the Pain Experience Scale (German: Schmerzempfindungsskala, SES). The painDETECT questionnaire quantifies the neuropathic component of pain. A higher score depicts a higher likelihood of neuropathic pain being present (Freynhagen et al., 2006).

The SES assesses the (a) affective and (b) sensory dimension of pain. Furthermore, information on (c) rhythmicity, (d) local depth, and (e) temperature are quantified as sub-scales of the sensory dimension of pain (Geissner, 1995).

#### Cognition

The Color-Word Interference Test (Stroop) is a frequently used test to quantify executive functioning. More specifically, the selective allocation of attention is rated (Lamers et al., 2010). The participants (a) must read color names, (b) name ink colors (no writings), and (c) name ink colors of words. However, in this last condition, the ink color and the color words do not match (incongruent stimulus). As outcomes, we used the time needed for condition c (incongruent stimulus) as well as the time difference of conditions b & c (time costs of the incongruent stimulus). Both outcomes are commonly assessed (Uttl and Graf, 1997).

#### Fear of Falling

We rated fear of falling using the Falls Efficacy Scale International (FES-I) (Dias et al., 2006). The questionnaire assesses the subjective fear to fall during various common daily activities. The German version of the FES-I was validated and depict good quality criteria (Delbaere et al., 2010). As the outcome, a single value reflecting the amount of fear of falling was calculated.

#### Depression

To register depression symptoms, we deployed the German version of the Center for Epidemiologic Studies-Depression Scale (CES-D) for screening purposes. This validated questionnaire is suitable for a self-assessment of depressive symptoms in the general population (Meyer and Hautzinger, 2001; Lehr et al., 2008). High outcome scores indicate a high probability of having a depression.

#### Health Status

The overall health status was assessed with the 12-Item Short-Form Health Survey (SF-12 Ware et al., 1996). Based on that 12 items a physical component summary, as well as a mental health summary score, were analyzed. High scores are interpreted as a good health status.

#### Peripheral Sensation

Mechanical and vibration detection thresholds were assessed as described in the “quantitative sensory testing” battery (Rolke et al., 2006). To test the mechanical detection threshold, we used Frey filaments (0.5, 1, 2, 4, 8, 16, 32, 64, 128, 256, 512 mN; MARSTOCKnervtest, Schriesheim, Germany). The force was applied to the lateral malleolus of the dominant foot. A series of descending, and ascending stimulus intensities was applied. The threshold was calculated as the geometric mean from five repetitions.

To test the vibration detection threshold, the Rydel–Seiffer graded tuning fork (64 Hz, 8/8 scale) was applied at a proximal elevation of the tibia (tibia tuberositas). The participants were asked to indicate once they did not feel any vibrations anymore. The threshold was then defined as the mean of three repetitions.

#### Proprioception

Proprioception was tested with an inertial sensor-based reproduction test using an active-active procedure as described by Arvin et al. (2015). To measure the knee angle in real-time using an in-house software, inertial sensors (MTw2, Xsens Technologies B.V., Enschede, The Netherlands, sampling rate: 100 Hz) were fixed to the dominant shank (medial and distal to the tibia tuberositas) and to the iliotibial tract at the middle of the thigh. The participants stood on a wooden platform keeping their eyes closed. The participants were then asked to slowly flex their knee. The examiner said “stop” once a knee flexion angle of about 40° was reached. Deviations from the target angle up to ±5° were allowed and not corrected to reduce fatiguing effects in the older adults. The participants memorized the knee angle, returned to the start position and reproduced that angle. The mismatch error was registered. This procedure was repeated 10 times, but the first two times were considered learning trials and, thus, not analyzed. As outcomes, we calculated the mean of the absolute error and the standard deviation of the signed error.

#### Physical Activity

The “Freiburger Fragebogen zur körperlichen Aktivität” (FFkA Frey et al., 1999) is a validated instrument to register health-related physical activity (PA, in hours per week) during the last seven days. Since physical activities generally follow a yearly seasonal pattern (Cepeda et al., 2018), we adjusted, where appropriate, for this seasonal component using a sine/cosine regression with a yearly period. This seasonal regression model is often used in time series analysis and allows for an accurate description (*R*^2^ = 0.99) of the yearly PA mean pattern of old-elderly adults (aged ≥75 years) reported in Figure 1 of Cepeda et al. (2018). The data used to build and validate the seasonal regression model was extracted from Figure 1 in Cepeda et al. (2018) using the open source software Engauge Digitizer (version 10.7 by Mark Mitchell).

**Figure 1:**
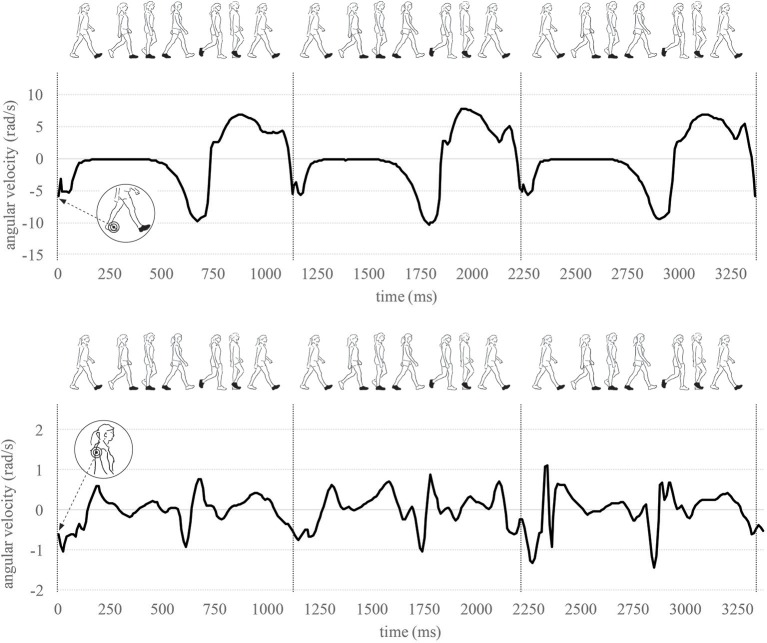
Sensor placement at the foot and trunk. As an example, one part of the sensor signal across three strides is illustrated.

### Statistics

The number of predictor variables (Table 2) was relatively large in comparison to the sample size and collinearities between predictor variables would cause variance inflations in the estimators of a classical regression analysis. Therefore, we used a principal component regression (PCR) analysis to reduce the dimension of the predictor space, to gain orthogonalized predictors and to link the identified principal components (PC) with the gait stability responses (primary outcomes) as well as the gait variability responses (secondary outcomes, **Table 4**). The number of factors was determined using the Kaiser criterion (percentage of variance explained: 75.3%).

To facilitate the interpretation, we used VARIMAX-rotated PCs and considered their largest (≥0.5) standardized factor loadings (Table 3). Gender and muscular fitness are included in the same factor (PC three). To get further insights into the relationship of gender and muscular fitness with the response variables, we analyzed the correlations (Pearson's r) between each of the muscular fitness outcomes (S2S_PeakV_ and S2S_MeanV_) and each of the gait stability and gait variability outcomes separately for male and female participants (**Table 5**). Additionally, we tested for gender differences in the gait stability and gait variability outcomes using *t*-tests for independent samples (**Table 6**).

**Table 3:** Results of the principal components analysis.

	**Principle components**										
		**1**	**2**	**3**	**4**	**5**	**6**	**7**	**8**	**9**	**10**
		**Pain**	**Balance**	**Strength &Gender**	**Physical activity**	**Cognition**	**Proprioception**	**Mental health**	**Osteoarthritis/ prosthesis**	**BMI**	**Periperal sensation**
	Gender			−0.79							
	Age										0.51
	BMI									0.77	
	Osteoarthritis								0.70		
	Prosthesis								0.68		
	S2S_PeakV_			0.91							
	S2S_MwV_			0.94							
Balance	Sway_dl,eo_		0.84								
	Sway_st,eo_		0.90								
	Sway_dl,ec_		0.90								
	Sway_st,ec_		0.87								
	Pain_neuro_ (Pain Detect)	0.64									
Pain SES	Pain_affect_	0.85									
	Pain_sens_	0.97									
	Pain_sens, rhythm._	0.65									
	Pain_sens, depth_	0.82									
	Pai_sens, temp._	0.75									
Stroop	Cog_ink_					0.97					
	Cog_relink_					0.95					
	FES-I										
	CES-D							0.78			
SF12	SF12_physical_									−0.62	
	SF12_mental_						−0.88				
QST	Sens_VibDT_										−0.66
	Sens_MechDT_										0.69
Prop.	Prop_MeanErr_						0.89				
	Prop_SDErr_						0.88				
FFKA	FFkA_basic_				0.73						
	FFkA_Extracurr_				0.60			0.54			
	FFkA_Sports_				0.58						
	FFkA_total_				0.95						

*The principal components analysis was used to reduce the dimension of the predictor space (Table 2) for a subsequent regression analysis (Table 4). To facilitate the interpretation, we used VARIMAX-rotated PCs and considered their largest (≥0.5) standardized factor loadings. Variable abbreviations are outlined in Table 2*.

## Results

For 90 participants, all outcome measures were analyzed. Test data of 12 participants could not be included due to technical problems of the inertial sensor system (*n* = 5) or some participants did not want to answer all questionnaires (*n* = 7). Since only 9 participants self-reported having diabetes, this factor was not included in the statistical analysis. Of the 90 participants analyzed, 41 (46%) reported having at least one fall and 19 (21%) having two falls within the last 12 months.

Pronounced seasonal patterns were found and corrected for the overall PA [*F*_(2, 99)_ = 8.62, *p* < 0.001], the basic PA [*F*_(2, 99)_ = 13.85, *p* < 0.001], and the sports-related PA [*F*_(2, 99)_ = 3.55, *p* = 0.03]. No significant seasonal pattern was found for the extracurricular PA.

The results of the principal components analyses are displayed in Table 3. Out of the 31 outcome measures, 10 factors were extracted in total: (1) “pain,” (2) “balance,” (3) “strength and gender,” (4) “physical activity,” (5) “cognition,” (6) “proprioception,” (7) “mental health,” (8) “Osteoarthritis and prosthesis,” (9) “BMI,” and (10) “peripheral sensation.”

The results of the regression analysis are depicted in Table 4. The factor “strength and gender” is a significant predictor for LDS foot (LDS_foot, ST_: *p* = 0.001, LDS_foot, DT_: *p* = 0.050) and trunk (LDS_trunk, ST_: *p* = 0.001, LDS_trunk, DT_: *p* < 0.001). A higher relative muscular fitness and/or being male correlated with a lower largest Lyapunov exponent (better LDS). Furthermore, more physical activity improved LDS of the foot during single (*p* = 0.006) and dual-task walking (*p* = 0.040) as well as to LDS of the trunk during single task walking (*p* = 0.007). A low peripheral sensation diminished the foot LDS (LDS_foot, ST_: *p* = 0.002, LDS_foot, DT_: *p* < 0.001) but there was not even a trend regarding the LDS of the trunk. BMI (LDS_foot, ST:_ *p* = 0.043), pain (LDS_foot, DT:_ *p* = 0.030) and the factor “Osteoarthritis and prosthesis” (LDS_trunk, DT:_ *p* = 0.006) were each a significant predictor but only in one model. Good balance abilities (*p* = 0.094) and a lower BMI (*p* = 0.075) tended to improve LDS_foot, DT_.

**Table 4:** Regression analyses were used to link the identified principal components (Table 3) with the gait stability responses (primary outcomes) and the gait variability responses (secondary outcomes).

	**Primary analysis**								**Secondary analysis**							
	**LDS foot**				**LDS trunk**				**SD Stride length**				**SD stride time**			
	**ST**		**DT**		**ST**		**DT**		**ST**		**DT**		**ST**		**DT**	
	**beta**	**(*p*)**	**beta**	**(*p*)**	**beta**	**(*p*)**	**beta**	**(*p*)**	**beta**	**(*p*)**	**beta**	**(*p*)**	**beta**	**(*p*)**	**beta**	**(*p*)**
Pain	0.155	(0.101)	0.214	(0.030)	−0.054	(0.593)	0.073	(0.462)	−0.033	(0.757)	−0.040	(0.700)	0.239	(0.019)	0.203	(0.058)
Balance	0.099	(0.297)	0.165	(0.094)	−0.162	(0.113)	−0.062	(0.532)	0.254	(0.020)	−0.016	(0.880	0.095	(0.348)	−0.004	(0.973)
Strength & Gender	−0.318	(0.001)	−0.188	(0.050)	−0.331	(0.001)	−0.432	(< 0.001)	0.101	(0.338)	0.111	(0.277)	−0.207	(0.039)	−0.107	(0.305)
Physical activity	−0.260	(0.006)	−0.197	(0.040)	−0.272	(0.007)	−0.103	(0.287)	−0.124	(0.237)	−0.256	(0.014)	−0.255	(0.011)	−0.318	(0.003)
Cognition	−0.049	(0.597)	−0.116	(0.226)	0.025	(0.801)	−0.012	(0.902)	0.112	(0.286)	0.280	(0.007)	−0.047	(0.635)	−0.022	(0.833)
Proprioception	−0.014	(0.882)	−0.141	(0.144)	−0.005	(0.962)	−0.006	(0.950)	0.148	(0.162)	−0.022	(0.831)	0.177	(0.077)	−0.019	(0.855)
Mental health	0.105	(0.257)	−0.063	(0.518)	0.123	(0.216)	−0.113	(0.255)	−0.087	(0.410)	0.146	(0.155)	0.117	(0.240)	0.095	(0.370)
Osteoarthritis/prosthesis	−0.023	(0.800)	−0.032	(0.734)	0.135	(0.175)	0.273	(0.006)	0.064	(0.540)	−0.163	(0.111)	0.007	(0.941)	−0.098	(0.343)
BMI	0.191	(0.043)	0.172	(0.075)	0.098	(0.327)	−0.065	(0.501)	0.062	(0.557)	−0.112	(0.275)	0.137	(0.173)	−0.009	(0.933)
Periperal sensation	0.293	(0.002)	0.430	(< 0.001)	−0.001	(0.995)	0.150	(0.123)	0.032	(0.763)	0.034	(0.740)	−0.030	(0.764)	0.216	(0.039)
Adjusted R^2^	0.255		0.260		0.150		0.233		0.042		0.123		0.148		0.116	

*ST, Single-task walking; DT, Dual-task walking; dark gray, p < 0.05 indicating a significant effect; light gray, 0.05 ≤ p < 0.01 indicating a non-significant tendency*.

Within the secondary analysis, Stride-to-stride gait variability was analyzed. Again, “strength and gender” (SD_StrideTime, ST_: *p* = 0.039) and “physical activity” (SD_StrideLength, DT_: *p* = 0.014, SD_StrideTime, ST_: *p* = 0.011, SD_StrideTime, DT_: *p* = 0.003,) were significant predictors. “Balance” (SD_StrideLength, ST:_ *p* = 0.020), “pain” (SD_StrideTime, ST_: *p* = 0.019), “cognition” (SD_StrideLength, DT_: *p* = 0.007) and “peripheral sensation” (SD_StrideTime, DT_: *p* = 0.039) were only in one model of the secondary analysis significant predictors. Good “proprioception” or less “pain” tended to improve SD_StrideTime, ST_ (*p* = 0.077) and SD_StrideTime, DT_ (*p* = 0.058), respectively.

Knowing, that muscular fitness and gender were included into one factor, we determined the relation of muscular fitness (outcomes S2S_PeakV_ and S2S_MwV_) with the gait stability and gait variability outcomes for men and women, separately (Table 5). For men, a higher muscular fitness improveed LDS_foot, ST_ (S2S_PeakV_: *r* = −0.33, *p* = 0.010), LDS_foot, DT_ (S2S_PeakV_: *r* = −0.30, *p* = 0.019) and SD_StrideLength, ST_ (S2S_PeakV_: *r* = −0.24, *p* = 0.049; S2S_MeanV_: *r* = −0.25, *p* = 0.043). Furthermore, S2S_PeakV_ (LDS_trunk, DT_: *r* = −0.21, *p* = 0.077) and S2S_MeanV_ (LDS_foot, ST_: *r* = −0.23, *p* = 0.052; LDS_foot, DT_: *r* = −0.19, *p* = 0.092) tended to increase LDS. For women, SD_StrideTime, ST_ was correlated with S2S_PeakV_ (*r* = −0.36, *p* = 0.005) and S2S_MeanV_ (*r* = −0.40, *p* = 0.002). LDS_trunk, DT_ (S2S_MeanV:_ *r* = −0.23, *p* = 0.059) tended to be correlated with muscular fitness measures.

**Table 5:** The correlations (Pearson's r) between each of the muscular fitness outcomes (S2S_PeakV_ and S2S_MeanV_) and each of the gait stability and gait variability outcomes were separately assessed for male and female participants.

		**Men (*n* = 49)**		**Women (*n* = 46)**	
		**S2S_**PeakV**_*r* (*p*)**	**S2S_**MwV**_*r* (*p*)**	**S2S_**PeakV**_*r* (*p*)**	**S2S_**MwV**_*r* (*p*)**
LDS foot	ST	−0.327 (0.010)	−0.232 (0.052)	−0.096 (0.256)	−0.185 (0.102)
	DT	−0.296 (0.019)	−0.193 (0.092)	0.013 (0.465)	−0.056 (0.356)
LDS trunk	ST	0.017 (0.455)	0.126 (0.192)	−0.094 (0.259)	−0.177 (0.111)
	DT	−0.207 (0.077)	−0.128 (0.191)	−0.129 (0.196)	−0.234 (0.059)
SD Stride length	ST	−0.237 (0.049)	−0.246 (0.043)	−0.179 (0.110)	−0.191 (0.094)
	DT	0.070 (0.314)	0.034 (0.408)	0.043 (0.386)	0.039 (0.397)
SD Stride time	ST	−0.099 (0.246)	−0.130 (0.183)	−0.363 (0.005)	−0.397 (0.002)
	DT	−0.109 (0.227)	−0.118 (0.209)	−0.201 (0.091)	−0.219 (0.072)

*ST, Single-task walking; DT, Dual-task walking. dark gray, p < 0.05 indicating a significant effect; light gray, 0.05 ≤ p < 0.01 indicating a non-significant tendency*.

Gait stability and gait variability measures of male vs. female participants are given in Table 6. Men depicted better LDS of foot (LDS_foot, ST_: *p* = 0.017, *d* = 0.48; LDS_foot, DT_: *p* = 0.029, *d* = 0.45) and trunk (LDS_trunk, ST_: *p* < 0.001, *d* = 0.82; LDS_trunk, DT_: *p* < 0.001, *d* = 0.99) but higher gait variability (SD_StrideLength, ST_: *p* < 0.001, *d* = 0.85) than women.

**Table 6:** We tested for gender differences in the gait stability and gait variability outcomes using *t*-tests for independent samples (ST, single-task walking; DT, Dual-task walking).

				**Male**		**Female**		**Male vs. female**			
				**Mean**	**SD**	**Mean**	**SD**	***t***	**df**	***p***	***d***
Primary analysis	LDS foot		ST	1.58	0.15	1.66	0.17	−2.42	100	0.017	−0.48
			DT	1.72	0.19	1.81	0.22	−2.22	96	0.029	−0.45
	LDS trunk		ST	0.77	0.12	0.89	0.16	−4.12	100	< 0.001	−0.82
			DT	0.83	0.13	0.98	0.17	−4.91	96	< 0.001	−0.99
Secondary analysis	SD Stride length	[mm]	ST	29	7	24	5	4.30	100	< 0.001	0.85
		[mm]	DT	31	9	29	8	1.32	98	0.188	0.26
	SD stride time	[ms]	ST	17	7	17	5	0.04	100	0.967	0.01
		[ms]	DT	25	11	28	18	−0.76	96	0.447	−0.15

## Discussion

The aim of the cross-sectional study was to explore influencing intrinsic factors on local dynamic gait stability and gait variability in an older population. The four dimensions (factors of a principal component analysis) (1) “strength and gender,” (2) “physical activity,” (3) “pain,” and (4) “peripheral sensation” were each associated with at least two of the analyzed gait stability/variability measures. Dimension (5) “balance” was a significant predictor in only one gait measure. While dimension (6) “proprioception” tends to correlate with a gait variability measure, we did not find a dependency of mental health on any gait measure. Hereafter, we will discuss these dimensions one after another:

1. The results suggest that participants with higher relative muscle performance or men walk more stable. Since both muscular fitness (sit-to-stand test) and gender were merged into one factor, we analyzed their individual contributions on gait stability and gait variability, separately. Comparing male and female participants, we observed significant differences in gait stability. Regarding the stability measures of the primary analysis, men walk more stable than women. This could be a reason why women are more likely to fall (WHO, 2008; Robinovitch et al., 2013). Despite this, the correlation of relative muscle performance with these primary measures was, on the one hand, stronger in men than in women regarding the gait stability measures but on the other hand, more pronounced in women as compared to men regarding the gait variability measures. Overall, the strongest correlations depicted only medium effects. The gait measures used in the current study reflect the system's capacity to recover from small perturbations (i.e., neuromuscular noise and wind, Bruijn et al., 2013). However, other types of stability, such as the recovery from larger perturbations (e.g., after tripping), might require more strength. This would also explain the comparatively low effect sizes of the correlation analysis. This result suggests that lower extremity muscular fitness is less relevant for LDS but could be more relevant for other kinds of gait stability (e.g., recovery from larger perturbations). This is in line with the recommendation to include strength exercises into fall prevention programs (Sherrington et al., 2011).
2. The amount of physical activity is also a strong predictor of most gait stability and gait variability measures during both, single-task and dual-task walking. This finding is in agreement with the broad evidence for beneficial effects of regular physical activity for enhancing and maintaining older adults' fitness as well as mental and physical health-related quality of life (Taylor et al., 2004; Netz et al., 2005; Nelson et al., 2007).
3. In our study, pain predicted a few gait outcomes. This is in line with previously reported data (Hamacher et al., 2016). In the paper of Hamacher et al. dual-task costs of gait depended on pain severity. In fact, pain is known to affect muscle activity and biomechanical behavior (Hodges, 2011) and pain disrupt cognitive functions and executive control (Keogh et al., 2013). Thus, it is surprising that the effects of pain on gait stability or gait variability were not more pronounced. A reason could be that the pain questionnaires were not assessed during the test day of the gait analysis which could have reduced the measured effect of pain on gait stability or variability measures.
4. Peripheral sensation was a significant predictor of foot LDS. In another study, touch and vibration sense were correlated with static balance performance (Lord et al., 1991) confirming our results. Interestingly, peripheral sensation did primarily effect foot LDS but not trunk LDS. In further studies, phase-dependent local dynamic stability (Ihlen et al., 2015) should be used to reveal if such effects are restricted to the stance or swing phase of gait.
5. We did not reveal any significant effect of balance in the primary analysis and only one within the secondary analysis. This is surprising since, in most studies, balance deficits being discussed to be a relevant risk factor for falls (Lord et al., 2003; Ambrose et al., 2013; Pfortmueller et al., 2014) and exercises to improve balance have been suggested to be included into fall prevention programs (Sherrington et al., 2008, 2011). Our results could imply that (static) balance is not that important for gait as it is for preventing falls in general (e.g., during quiet standing). It is known that posture control concepts are fundamentally different for standing and walking (Winter, 1995). Furthermore, it is known, that balance abilities are context-specific (Sibley et al., 2015; Kümmel et al., 2016). Our result could also be a limitation of the methods chosen. We only assessed static balance measures but no dynamic balance parameters and it is known that the predictive value for falls depends on the method (Muir et al., 2010) and that there is a high intrasubject variability in individual concepts maintaining postural stability (Pasma et al., 2014).
6. We did not find a significant dependency of the active joint position sense on gait stability or gait variability. This is in contrast with the result of Lord et al. (1991), who reported a relation of proprioception and static and dynamic balance measures. Non-questionable, proprioception plays a key role in motor control and functional joint stability (Riemann and Lephart, 2002; Proske and Gandevia, 2012) but the quality criteria (reliability and validity) have been questioned, in general (Riemann et al., 2002; Benjaminse et al., 2009; Hillier et al., 2015). Low quality criteria could explain the missing relationship between proprioception and gait stability or gait variability.

Overall, the explained variance of the regression model is rather low (adjusted *R*^2^ range from 0.04 to 0.26). In models predicting static or dynamic balance, the multiple *R* ranged from 0.24 to 0.37 (Lord et al., 1991) which would be an *R*^2^ of 0.06 to 0.14. These *R*^2^ values are comparable to ours (between 0.15 and 0.26 for the models predicting LDS of the foot and trunk, Table 4). However, the low explained variance highlights the fact that there could be more relevant factors for gait stability and gait variability (as well as for balance) that were not addressed, yet. However, a strength of our study is the large number of considered parameters. Furthermore, the study sample seems to be sufficient to reveal effects relevant to the practice. On the other hand, this is a cross-sectional study. Thus, the results should be confirmed with experimental designs, for example with intervention studies designed to improve gait stability. Additionally, we did not assess vision, vestibular functioning, peripheral nerve tests and reflexes or Vitamin D deficiency and other variables that also have been discussed to be relevant risk factors for falls (Tinetti et al., 1988; Lord et al., 2003; Ambrose et al., 2013; Pfortmueller et al., 2014). At last, we assessed the participants' ability to recover from small perturbations. There are other types of gait stability that should be addressed in further studies.

In conclusion, the participants' ability to recover from small perturbations (as measured with the largest Lyapunov exponent) seems to be related to (1) gender and muscular fitness, (2) the amount of physical activity the participants spent every week, (3) peripheral sensation (mechanical and vibration detection threshold), and (4) pain status. No or minor effects were found for balance, proprioception, cognition or mental health. Since the explained variance is still rather low, there could be more relevant factors that were not addressed, yet.

## Author Contributions

DH and AZ planned and designed the study. DH, CH, VH, CK, and TT collected the data. DL and DH analyzed the data. DH, DL, and AZ drafted the manuscript. All authors critically revised the manuscript. All authors approved the final version of the manuscript.

### Conflict of Interest Statement

The authors declare that the research was conducted in the absence of any commercial or financial relationships that could be construed as a potential conflict of interest.
